# Synaptic plasticity in hippocampal CA1 neurons and learning behavior in acute kidney injury, and estradiol replacement in ovariectomized rats

**DOI:** 10.1186/s12868-019-0534-4

**Published:** 2019-10-04

**Authors:** Fatemeh Sharifi, Parham Reisi, Maryam Malek

**Affiliations:** Department of Physiology, School of Medicine, Isfahan University of Medical Sciences, Isfahan, Iran

**Keywords:** Synaptic plasticity, Hippocampus, Learning and memory, Acute kidney injury, Estradiol replacement, Ovariectomized rats

## Abstract

**Background:**

Neurological complications may occur in patients with acute or chronic renal failure; however, in cases of acute renal failure, the signs and symptoms are usually more pronounced, and progressed rapidly. Oxidative stress and nitric oxide in the hippocampus, following kidney injury may be involved in cognitive impairment in patients with uremia. Although many women continue taking hormone therapy for menopausal symptom relief, but there are also some controversies about the efficacy of exogenous sex hormones, especially estrogen therapy alone, in postmenopausal women with kidney injury. Herein, to the best of our knowledge for the first time, spatial memory and synaptic plasticity at the CA1 synapse of a uremic ovariectomized rat model of menopause was characterized by estradiol replacement alone.

**Results:**

While estradiol replacement in ovariectomized rats without uremia, promotes synaptic plasticity, it has an impairing effect on spatial memory through hippocampal oxidative stress under uremic conditions, with no change on synaptic plasticity. It seems that exogenous estradiol potentiated the deleterious effect of acute kidney injury (AKI) with increasing hippocampal oxidative stress.

**Conclusions:**

Although, estrogen may have some positive effects on cognitive function in healthy subjects, but its efficacy in menopause subjects under uremic states such as renal transplantation, needs to be further investigated in terms of dosage and duration.

## Background

Acute kidney injury (AKI) with considerable morbidity and mortality rates is a common complication of critically ill patients [1]. Despite advances in supportive and palliative care, patients with AKI continue to experience progressive worse outcomes. Although, most therapeutic strategies of AKI have focused on intra-renal outcomes, but extra-renal manifestations are responsible for the development of AKI, thereby resulting in death [2]. The crosstalk of kidney with other organs by causing multi-organ dysfunction complicates and restricts therapeutic approaches [3]. Uremic encephalopathy is a neurological syndrome that may occur in patients with acute or chronic renal failure; however, in patients with acute renal failure, the signs and symptoms are usually more pronounced, and progressed rapidly [4, 5]. The symptoms can vary widely from fatigue and cognitive impairment to coma [6]. It appears that oxidative stress and nitric oxide (NO), through reaction with reactive oxygen species and formation of peroxynitrite (a highly reactive and cytotoxic byproduct), can be involved in the pathogenesis of uremic encephalopathy under chronic renal injury states [7]. Oxidative damage to the brain tissue and its substructures may explain the relationship between increased hippocampal oxidative stress and cognitive impairment in uremia. In addition, the severity of the outcomes reportedly varied between males and females, following acute or chronic renal failure [8, 9]. There is also a controversy about the effect of estrogens on renal damage to ovariectomized animals, as well as exogenous sex hormones in postmenopausal women [10]. While some reports indicated more deterioration by administration of estrogen [11, 12], others reported an improvement of renal function [13, 14]. These disparities may be due to the differences between various animal models of renal injury or differences in the type, dosage or mode of estrogen treatment administered.

A high dose of estrogen therapy was accompanied by glomerular injury, reduced creatinine clearance and albuminuria in ovariectomized mice [15]. Estrogen also stimulates both the renin–angiotensin system and angiotensinogen that may exert specific deleterious effect on progression of renal disease [16, 17]. Karl et al. demonstrated that continuous estradiol replacement, but not intermittent, preserved glomerular function and structure in ovariectomized mice [18]. Estrogen efficacy on cognition also remains unknown in uremic menopausal patients. The discovery of estrogen receptors (ER-β and ER-α) in hippocampal cells has indicated how estrogen differently affects the inhibitory and excitatory synapses [19, 20].

Considering the importance of the hippocampus as a brain region, involved in learning and memory and its relationship with AKI, the aim of our study was to investigate the role of AKI and estradiol supplementation on the hippocampal cognitive function and synaptic plasticity in the animal model of menopause.

## Results

### Water maze

**Fig. 1:**
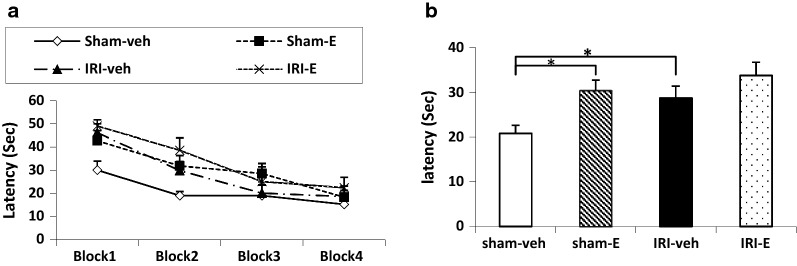
**a** Escape latency to reach the platform in each block (day) during four training days or acquisition trials. Each block is the average of four consecutive trials. **b** Cumulative acquisition trials of 4 days in different groups. Data are shown as mean ± SEM (n = 10). *P < 0.05

Global analysis revealed that latency to reach the platform was progressively and significantly reduced over the 4 days of training in all experimental groups, indicating spatial acquisition (Block effect, F (3, 102) = 34.86, *P *< 0.001, two-way repeated measures ANOVA). No significant differences in reduction pattern were observed between the groups during the acquisition of learning (Block and Group effect, F (9, 102) = 1.25, P = 0.26; Fig. 1a). This indicates that all groups learned over the 4 days of training. One-way ANOVA revealed a significant difference between sham-veh rats compared to other treatment groups at the beginning of learning procedure (block 1 and 2). Analysis of performance the animals in each trial within the block1 showed significant decreases in the latency to reach the platform of the first trial in sham-veh rats relative to treatment groups (*P *< 0.05).

In cumulative acquisition trials of 4 days (Fig. 1b), a statistically significant difference was found between AKI group in comparison to the sham (sham-veh) group (28.72 ± 2.7 s and 20.83 ± 1.82 s respectively; *P *< 0.05), and also between the sham treated with estrogen (sham-E), compared to those in sham-vehicle (sham-veh) rats (30.42 ± 2.33 s and 20.83 ± 1.82 s respectively; *P *< 0.05).

**Fig. 2:**
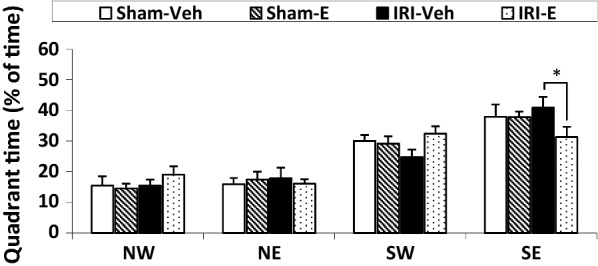
Percent time spent in each quadrant during the probe trial on the fifth day. The target quadrant is south-east (SE). Other quadrants are: north-west (NW), north-east (NE), and south-west (SW). Data are expressed as mean ± SEM (n = 10). *P < 0.05

Memory retrieval of rats in all groups was evaluated using a probe trial test. The percentage latencies of the rats within the target quadrant in a probe trial are shown on the fifth day in Fig. 2. All groups, except ischemic-estrogen treated (IRI-E) rats, spent a higher percentage of time in the target quadrant (SE), relative to the other non-target quadrants (Time effect, F (3, 34) = 475.115, *P *< 0.001). As shown, AKI rats treated with estrogen spent a significantly decreased percentage of time in the target quadrant during the memory retrieval, compared to the AKI rats (31.23 ± 3.4 s and 40.90 ± 3.6 s respectively; *P *< 0.05), indicating the destructive effect of estrogen in acute renal injury states. There was no difference in swimming performance between the experimental groups (data not shown).

### Short- and long-term synaptic plasticity

**Fig. 3:**
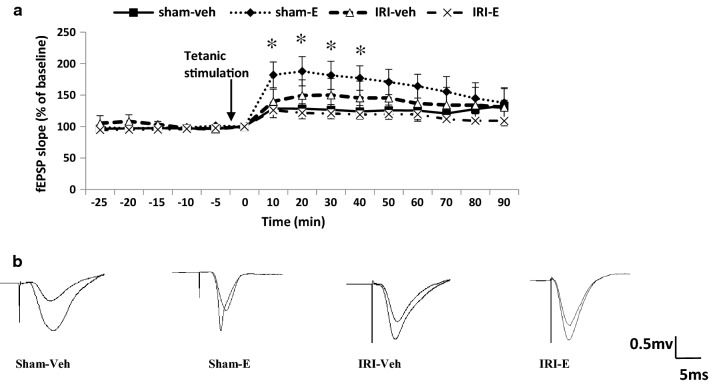
**a** Time-course of percentage change in the slope of long-term synaptic plasticity of CA1 neurons post-100 Hz tetanic stimulation of Schaffer collaterals. Data are presented as percentage change from baseline EPSP and expressed as mean ± SEM (n = 10). *P < 0.05 versus sham-veh group. **b** Sample traces show typical fEPSPs recorded in hippocampal CA1 neurons before and after LTP induction

As shown in Fig. 3, LTP was induced in the CA1 area of the right hippocampus by administering a train-of-four tetanus, delivered to the ipsilateral Schaffer collateral pathway. The short tetanus evoked an increase in the EPSP from the baseline that was nearly maintained for 90 min. Group data were collected from ten animals. A repeated measures ANOVA revealed that the average fEPSP slope declined over time in all groups (Time effect, F (8, 272) = 7.18, *P *< 0.001; Fig. 3a). Estradiol treatment induced temporary enhancement of EPSP slope (lasting 40 min after tetanic stimulation) in sham-operated animals (sham-E), compared to the sham vehicle treated group (sham-veh), and then diminished gradually (Time and Group effect, F (24, 272) = 2.16, *P *< 0.05; Fig. 3b).

**Fig. 4:**
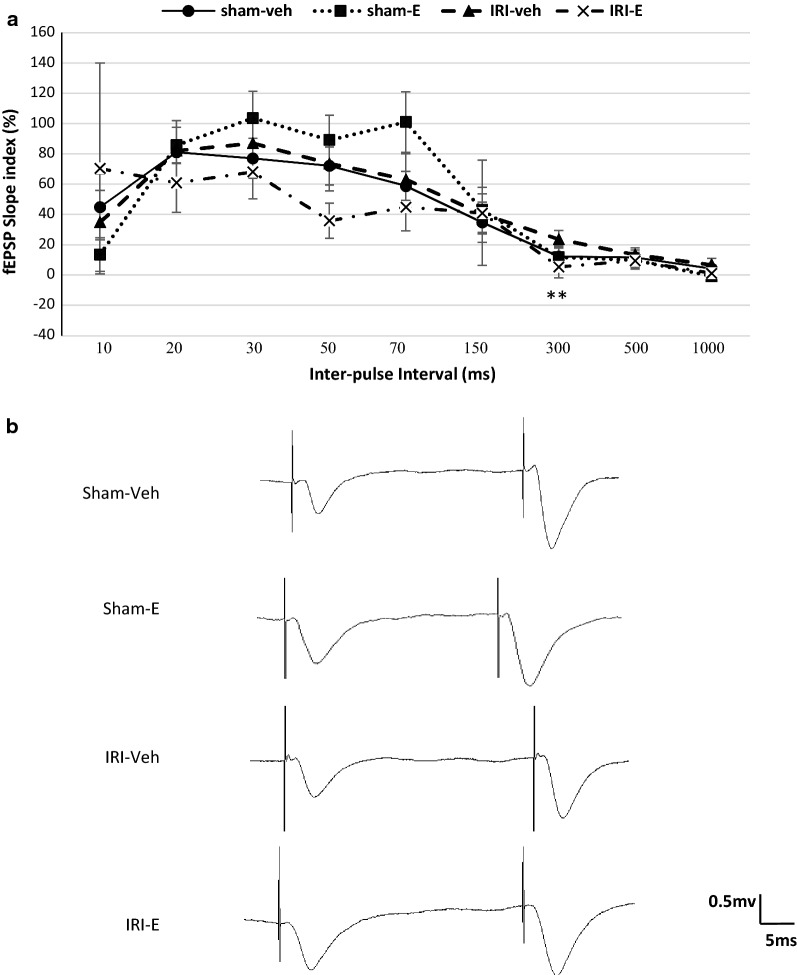
**a** Paired-pulse facilitation induction of fEPSPs recorded in the CA1 area after stimulation of Schaffer collaterals. The percentages denote the ratio of the second fEPSP slope to the first fEPSP slope. PPR (facilitation or depression) was tested at 10, 20, 30, 50, 70, 150, 300, 500 and 1000 ms IPIs between groups. Data are expressed as mean ± SEM (n = 10). **P < 0.01 IRI-E versus IRI-veh at the IPI 300. **b** Representative sample paired-pulse traces in experimental groups

Short-term plasticity was evaluated by paired-pulse ratios (the ratio of the second response to the first response), using a pair high similar stimulus intensity at different IPIs. Quantitative relationships between IPIs and the paired-pulse ratios of the fEPSP slope by the second pulse are shown in Fig. 4a.

No significant differences were found in the paired pulse ratios of the fEPSP slope between groups, except a minor depression in ischemic estradiol treated rats versus IRI-veh group at the IPI 300 (t (3.12) = 1.80, *P *< 0.01).

### Biochemical analysis

**Fig. 5:**
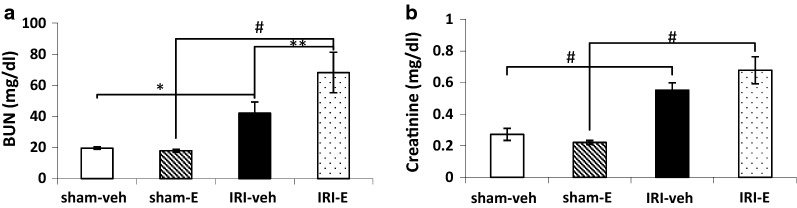
**a** Serum BUN and **b** creatinine levels at 24 h post-reperfusion. Data are shown as the mean ± SEM (n = 10). *P < 0.05, ** P < 0.01, and ^#^P < 0.0001

The serum nitrogen or blood urea nitrogen (BUN) and creatinine levels, as indicators of kidney function, are shown in Fig. 5a, b. Bilateral kidney IRI induced an increase in serum BUN (sham-veh versus IRI-veh (t (2.93) = 107.2, *P *< 0.05), and sham-E versus IRI-E (t (3.39) = 284.78, *P *< 0.001)), and creatinine levels (sham-veh versus IRI-veh, (t (4.67) = 1.42, *P *< 0.001), and sham-E versus IRI-E (t (5.21) = 39.77, *P *< 0.001), 24 h after reperfusion. Moreover, there were high BUN and creatinine levels in the estradiol-treated IRI group versus the vehicle-treated group (IRI-E versus IRI-veh, (t (1.76) = 3.26, *P *< 0.01, for BUN). However, the high rate of creatinine was not statistically significant between these treated groups.

**Fig. 6:**
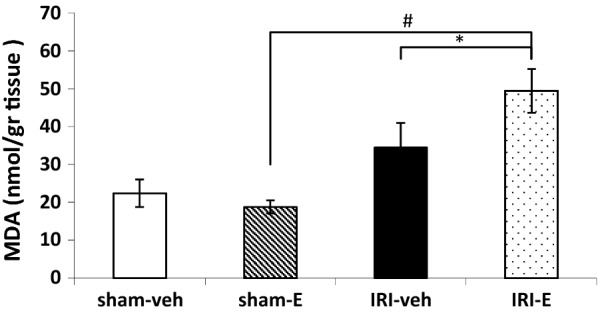
MDA concentration in the hippocampus of different study groups. Values are given as mean ± SE for each group (n = 10). *P < 0.05, and ^#^P < 0.0001

The effect of AKI and estrogen on the oxidative status of the hippocampus is shown in Fig. 6. Administration of estrogen in the IRI animals increased hippocampal MDA content as a marker of lipid peroxidation, compared to the vehicle-treated group (t (1.71) = 1.43, *P* < 0.05). Interestingly, although AKI was not able to increase hippocampal MDA content in the ischemic vehicle groups (IRI-veh versus sham-veh), but it showed a remarkable increase in the MDA level when combined with estrogen (t (4.84) = 12.23, *P *< 0.001; IRI-E versus sham-E). No statistically significant differences were found among other study groups.

**Fig. 7:**
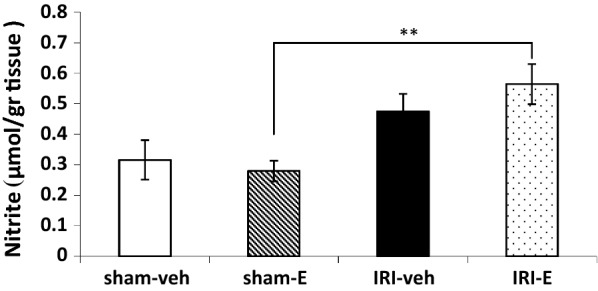
The nitrite concentration in the hippocampus tissue of four groups. Data are expressed as the mean ± SEM (n = 10). **P < 0.01

Moreover, hippocampal nitrite (the oxidation product of NO) levels were assessed to determine whether hippocampal NO levels were affected by AKI and estrogen. Although bilateral ischemia reperfusion did not significantly alter the nitrite level in vehicle-treated groups (IRI-veh versus sham-veh), but it presented reliable differences in estrogen treatment groups (t (3.67) = 4.21, *P* < 0.01; IRI-E versus sham-E). Statistical comparison of data presented no reliable differences between the other groups (Fig. 7).

## Discussion

The present study, for the first time to our knowledge, aimed at investigating behavioral and electrophysiological performance of ischemic AKI and estradiol replacement, in the ovariectomized rat model of menopause. In terms of behavioral results, the pattern of reduction in escape latency during block 1 to 4 in acquisition of learning was the same between the groups, as shown in Fig. 1a. Cumulative acquisition trials of 4 days at the first glance support the notion that ischemic AKI and estradiol treatment in ovariectomized sham rats may impair spatial learning capabilities, with no effect on the retention of spatial memory. This was confirmed with a higher overall latency to find the platform in the learning phase; there was no statistical difference in the spatial memory retrieval phase of these groups, compared to the related controls in the MWM test. However, because of the similar reduction pattern in latency toward the end of training in all groups, it appears that learning performance or strategies may be involved, rather than learning ability. One possibility is that estrogen signaling may interact with the dopaminergic system, through ERβ activation to change cognitive strategies [21, 22]. Furthermore, ischemic and estradiol-treated rats showed higher latency in the beginning of the learning procedure (block 1 and block 2), compared to the sham group. It appears that the statistical difference in Fig. 1b is not a result of learning impairment, but from baseline differences in latency in block 1 and 2 especially in trial 1. The difference observed in the first trial between sham and treated groups may be due to the influence of therapeutic interventions on the initial performance of the animals not learning capabilities.

Further training in blocks after the first and second days compensated the learning level of treatment groups and reached them to the learning level of the sham group on days 3 and 4, as seen in Fig. 1a. In other words, ischemic or treated rats needed more training to reach the learning level of the sham group, as learning was induced over a longer period than the sham group.

Globally, our behavioral data indicated that estradiol replacement might impair memory retrieval under uremic states without any significant effect in the sham-E group (Fig. 2). These results are in conflict with the literatures, which demonstrated an increase performance on MWM in healthy adult OVX rats [23, 24]. These discrepant results may be due to various variables such as estradiol dose, the timing of hormone therapy, age, the type of estrogen used, route of administration and the duration of hormone deprivation prior to treatment with estrogen [25, 26]. This study was conducted on a supra-physiological dose of estradiol (200 μg/kg, ip). El-Bakri et al. demonstrated that low dose of estrogen treatment in ovariectomized rats has a better outcome in spatial memory performance than the high dose [27]. An altered pharmacokinetic profile of estrogen was observed in postmenopausal women with renal failure [28]. Anderson et al. suggest that women with kidney injury should receive at least a 50% reduction in estradiol doses, compared to healthy subjects [29]. Therefore, the toxic effects of estrogen in IRI-E group may be due to the decreased catabolism of estradiol, which was not observed in the sham group. Gibbs et al. demonstrated that there is a window of opportunity after the loss of ovarian function during which, hormone replacement can effectively prevent the cognitive decline [30]. It appears that estrogen may exert trophic or protective actions at low physiological concentrations, but harmful at pharmacological concentrations under inflammatory states [31]. Biochemical analysis was confirmed hippocampal oxidative stress in IRI-E rats, suggesting a possible mechanism of cognitive impairment observed in this group. By contrast, the obtained results from other groups revealed no changes in biochemical markers, which further confirmed their behavioral responses. In support of this, Karimi et al. indicated that 45 min renal ischemia cannot significantly affect the number of CA1 neurons and long-term synaptic plasticity of the CA1 hippocampal synapses, because of inadequate deleterious strength of this ischemia duration [32]. In addition, Liu et al. found that 60 min renal ischemia has much more deleterious effects on cerebral structures than 45 min ischemia [33]. However, interestingly, AKI could not induce a hippocampal oxidative stress and increased nitrite content, but it induced increased oxidative stress and nitrite levels in the hippocampus when treated with a combination of estradiol. In the other words, exogenous estradiol potentiated the deleterious effect of AKI. Estrogen could have a pro-inflammatory role depending on a variety of criteria [34] and stimulate T cell-dependent immune responses in inflammatory situations such as seen in AKI [35]. Estrogen also is implicated in the activation of eNOS and nNOS, as well as the regulation of expression of all three nitric oxide synthase (NOS) isoforms in the immune, cardiovascular, and central nervous systems [36–38]. A comparison of plasma urea levels between groups demonstrated an adverse effect of estrogen on kidney function in AKI. This finding is in contrast to those reported a renoprotective role for estrogen [39–41]. The conflicting results of hormonal replacement therapy on the kidney remain unclear. The timing and concentration of estrogen administered after OVX may contribute to the variable outcome of hormone therapy after menopause [15]. Estrogen also activates both the renin-angiotensin system and angiotensinogen that may exert specific deleterious effect on progression of renal disease [16, 17].

In terms of electrophysiology, estradiol indicated a transient increase in the slope of LTP, which lasted 40 min after tetanic stimulation in sham-operated rats. Transient increase in fEPSP, following tetanization possibly reflects a transient activation of NMDA receptors, rather than protein synthesis. This result is consistent with the beneficial effects of estrogen on LTP in ovariectomized rats [42], but the effect was transient. It seems that estrogen dosage used in this study modulates short-term potentiation in the hippocampus under non-uremic conditions. This result may give further justification for no effect of estrogen on long-term spatial memory, as seen in the sham group. Indeed, we showed that estrogen induced hippocampal oxidative stress under uremic conditions, with some behavioral signs, and no change on long-term synaptic plasticity. It appears that the reduced expression of ERβby pro-inflammatory cytokines in AKI condition [43] results in a lower impact of estrogen on synaptic plasticity than the non-uremic states, which may also be manifested by a decline in spatial memory as described earlier.

It is now well-established that in many synapses, repeated stimuli delivered at short time intervals, lead to a transient decrease in postsynaptic potentials called short-term synaptic depression [44]. In contrast, an increase in the presynaptic calcium influx could increase the probability of release and contribute to facilitation or presynaptic short-term plasticity. Paired-pulse ratio reflects synaptic efficacy through the release probability of the presynaptic cell. Synapses with a low release probability are more likely to exhibit higher paired-pulse ratio, compared to those with a high release probability [45, 46]. Increased basal release probability, by elevating extracellular Ca^2+^ concentration led to a reduction in the paired-pulse ratio. In the present study, we have assessed neurotransmitter release probability at Schaffer collateral-CA1 synapses with the paired-pulse paradigm. For the Schaffer collateral synapses on CA1 pyramidal cells, paired-pulse plasticity varies considerably among the synapses in a correlated manner. The heterogeneity and variations in paired-pulse plasticity among the Schaffer collateral synapses are based on the following two factors: number of immediately releasable vesicles and the vesicle release probability [47]. Paired-pulse depression at some longer IPIs may reflect alterations in local circuitry and synaptic initial release characteristics of estradiol treatment in uremia [47]. No significant differences in paired-pulse facilitation were generally observed between experimental groups at different inter-pulse intervals. Taken together, this observation suggests that estrogen does not change the possibility of release of neurotransmitters from hippocampal presynaptic terminals or short-term synaptic plasticity, under the present experimental conditions.

## Conclusions

However, estrogen may have some positive effects on cognitive function in healthy menopausal subjects, but its efficacy in kidney injury, such as renal transplantation needs to be further investigated, in terms of dosage and duration. Although this animal model does not recapitulate all aspects of menopausal women, but our present findings require further investigation of the effects of estrogen in this regard. Future studies also will be required to determine the effects of progesterone in combination with estrogen.

## Methods

### Subjects

Experiments were carried out in adult female Wistar rats (Pasteur Institute, Tehran, Iran), weighing 200–250 g with an average age of 8 weeks. The rats were housed four per cage, under controlled environmental conditions (light on from 7:00 a.m. to 7:00 p.m.) and the temperature (22 ± 3 °C), with ad libitum access to tap water and standard laboratory diet. During this study, all animals were treated in accordance with the ethical guidelines for the Care and Use of Laboratory Animals (1996, published by National Academy Press, 2101 Constitution Ave. NW, Washington, DC 20055, USA), and approved by the Isfahan University of Medical Sciences Ethical Committee.

### Experimental groups

Forty ovariectomized rats were randomly assigned into four experimental groups, with ten animals in each group: sham-operated control group treated with estradiol (sham-E), ischemia–reperfusion injury group treated with sesame oil (IRI-veh), ischemia–reperfusion injury group treated with estradiol (IRI-E), and sham-operated control group treated with sesame oil (sham-veh).

### Experimental procedures

**Fig. 8:**
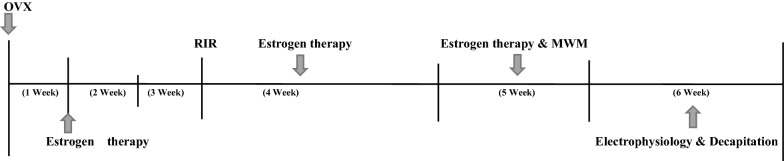
The study experimental design

A schematic illustration showing timelines for the complete experimental design was depicted in Fig. 8.

#### Ovariectomy and estrogen replacement

A week before the beginning of the study, the rats underwent bilateral ovariectomy (OVX) under anesthesia with an intraperitoneal injection of a mixture of xylazine (10 mg/kg) and ketamine (90 mg/kg). Briefly, a small midline abdominal incision was made to expose the uterus; the Fallopian tubes were excised; and the ovaries were clamped, tied, and removed. Finally, the skin was sutured with 3.0 silk.

The estradiol replacement therapy group received estradiol valerate subcutaneously (200 µg/kg; Abouraihan Pharmaceutical Co) [48], once a week for 4 weeks, commencing at 1 week after OVX surgery. The rats in the control group were injected with the same volume of sesame oil as that of the vehicle group once a week.

#### AKI model

AKI was induced by renal IRI during the hormone replacement therapy (2 weeks after injections). The anesthetized rats (ketamine–xylazine combination) were subjected to bilateral small flank incisions, the kidneys were exposed, and renal pedicles were occluded, using non-traumatic vascular clamps for 45 min. Following 45 min of ischemia, the clamps were removed, and successful revascularization of the kidney was confirmed visually. The muscle and skin layers were then sutured, and rats were allowed to recover. Blood was collected from the tail vein 24 h after reperfusion for renal function assessment [49].

### Behavior assessment

#### Morris water maze (MWM) test

Cognitive outcome was determined after recovery by the Morris water maze (MWM) test for five consecutive days. The water maze was a circular black-painted metal tank (180 cm in diameter) that was filled with water (21 ± 2 °C), and surrounded by various spatial cues. In brief, rats were trained individually to find the invisible submerged escape platform for four trials per day, with an inter-trial interval of 30 s for four consecutive days (four blocks, each block consisting of four trials). In each trial, the animal was released randomly from one of the four tank locations around the pool (north, south, east, and west) and allowed to freely swim until the hidden platform was found or 60 s elapsed for guiding on the platform, located in the southeast quadrant. The rats remained on the platform for 30 s before being taken off. The escape latency to reach the platform was recorded during sessions, by a video camera mounted above the tank. To evaluate memory retrieval, on the fifth day, the platform was removed and time spent in the target quadrant was evaluated, and compared in a 60 s trial as a probe trial [50].

### Electrophysiological study

#### Field excitatory postsynaptic potential (fEPSP)

After achieving deep anesthesia with urethane (1.6 g/kg, i.p.), the animal’s head was fixed, using a stereotaxic frame, and the surface skull was exposed. A bipolar stimulation microelectrode (stainless steel, 0.125 mm in diameter; Advent, UK) was inserted in the Schaffer collateral pathway of the right hippocampus (AP, 3; ML, 3.5; DV, 2.8–3), and a unipolar recording microelectrode was lowered into the ipsilateral CA1 area (AP, 4.1; ML, 3; DV, 2.5). The Schaffer collateral pathway was stimulated, and the extracellular field potential was recorded from the right CA1. The intensity of test stimuli after *I*/*O* function recording was adjusted to yield approximately 50% of the maximum response of the fEPSP slope.

#### Paired‐pulse response (PPR)

To examine the presynaptic transmitter release potential or short-term synaptic plasticity, equal strength pairs of stimulus pulses were delivered at inter-pulse intervals (IPIs) of 10, 20, 30, 50, 70, 150, 300, 500, and 1000 ms. The ratio was quantified as an average of three subsequent responses of each inter-stimulus interval to the average of three previous responses.

#### Long‐term potentiation (LTP)

After acquiring a steady state baseline of fEPSPs for 30 min, long‐term potentiation (LTP) induction was done by applying 100 Hz high-frequency stimuli (HFS) (four trains of 50 pulses, 0.15 ms stimulus duration, and 10 s inter-burst intervals), as previously described. LTP was expressed by changing the initial slope of fEPSP, as a percentage of the initial baseline value that was monitored for 90 min after tetanic stimulation.

### Brain tissue fixation and sectioning

**Fig. 9:**
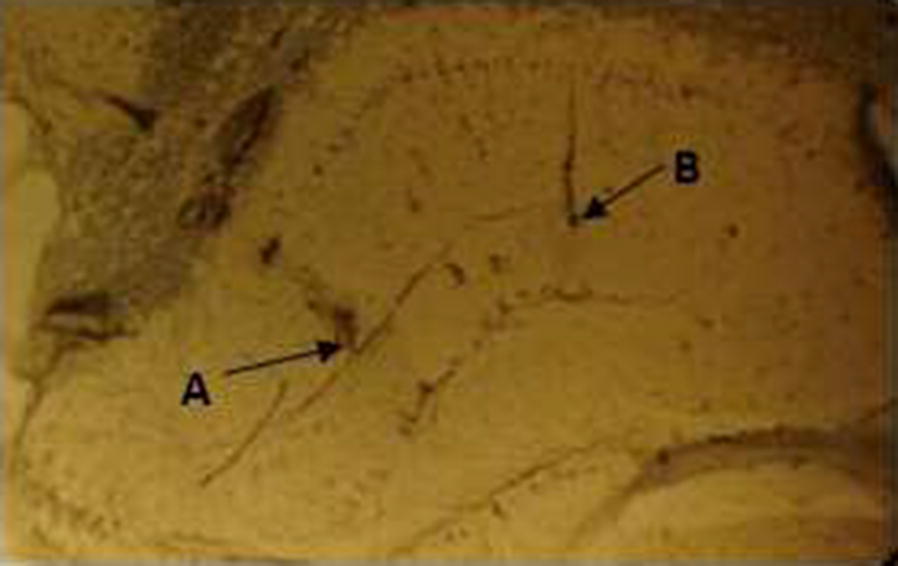
Coronal section of hippocampus showing tips of the stimulating and recording electrodes in the (B) Schaffer collateral pathway and (A) hippocampal area CA1

At the end of the study, rats were killed by decapitation under urethane anesthesia. The brains were rapidly removed, and implantation of electrodes in the correct location was determined by histological confirmation. For histological confirmation, the right hemisphere was excited and stored in 10% formalin solution for at least 10 days. Subsequently, electrode placement was verified by the obtained 5 μm-thickness transverse sections, using a freezing microtome. Sections were examined under a microscope and compared to the rat brain atlas [51] (Fig. 9). Then, the whole hippocampus was removed from the left hemisphere, and stored at − 70 °C for the biochemical analysis.

### Biochemical assessment

#### Renal function assessment

Kidney function was assessed 24 h post-reperfusion by measuring serum urea and creatinine (Cr). Blood samples (0.5 ml) were collected from the tail vein and centrifuged at 10,000×*g* for 3 min to separate the serum. The serum BUN and Cr levels were determined, using the quantitative diagnostic kits (Pars Azmoon, Iran).

#### Lipid peroxidation measurements

Malondialdehyde (MDA) levels were measured as an index of lipid peroxidation. MDA reacts with thiobarbituric acid as a thiobarbituric acid reactive substance (TBARS), and produces a pink species with a 532 nm absorption maximum [52]. Briefly, a mixture of 1 ml of 10% trichloroacetic acid, 1 ml of 0.67% thiobarbituric acid, and hydrochloric acid was added to 1.0 ml of the 10% (w/v) hippocampal homogenate, and the solution was mixed. The solution was heated for 45 min in a boiling water bath. After cooling, the solution was centrifuged at 1000×*g* for 10 min, and the absorbance of the supernatant was determined at 535 nm against a blank that contained all the reagents, except the sample. The amount of MDA was calculated as absorbance/1.65 × 10^5^.

#### Nitrite content determination

The nitrite level, a stable NO metabolite of the hippocampus was determined by the Griess reaction that has been described in detail elsewhere [53]. The 10% (w/v) homogenates were centrifuged at 1000×*g* for 10 min; the supernatants (100 µl) were mixed with the same volume of Griess reagent (1% sulfanilamide, 0.1% naphthalene diamine dihydrochloride, and 2.5% phosphoric acid), and this solution was incubated at room temperature for 10 min. Finally, the absorbance was determined at 520 nm. Nitrite concentration was determined from a standard nitrite curve generated, using NaNO_2_.

### Statistical analysis

Values are presented as mean ± SEMs, and analyzed by Student’s t tests or two-way analysis of variance (ANOVA), to confirm differences between groups. Post-hoc comparisons were made, using the Least Squares Design (LSD). Two-way repeated measures ANOVA was used to determine differences from the baseline after tetanic stimulation in the LTP experiment. An α-level of P < 0.05 was required for statistical significance.

## Data Availability

The datasets used and/or analyzed during the current study are available from the corresponding authors on reasonable request.

## Abbreviations

AKI: acute kidney injury
NO: nitric oxide
BUN: blood urea nitrogen
MDA: malondialdehyde
TBARS: thiobarbituric acid reactive substance
LTP: long-term potentiation
Cr: creatinine
HFS: high-frequency stimuli
PPR: paired-pulse response
IPIs: interpulse intervals
fEPSP: field excitatory postsynaptic potential
MWM: Morris water maze
OVX: ovariectomy
IRI: ischemia–reperfusion injury
Veh: vehicle
